# Thought Insertion as a Self-Disturbance: An Integration of Predictive Coding and Phenomenological Approaches

**DOI:** 10.3389/fnhum.2016.00502

**Published:** 2016-10-12

**Authors:** Philipp Sterzer, Aaron L. Mishara, Martin Voss, Andreas Heinz

**Affiliations:** ^1^Department of Psychiatry and Psychotherapy, Campus Charité Mitte, Charité – UniversitätsmedizinBerlin, Germany; ^2^Department of Clinical Psychology, Chicago School of Professional Psychology, Southern California CampusLos Angeles, CA, USA; ^3^Department of Psychiatry and Psychotherapy, Charité University Hospital and St. Hedwig HospitalBerlin, Germany

**Keywords:** self-disorders, first rank symptoms, schizophrenia, phenomenological psychiatry, Bayesian inference, predictive coding, Mayer-Gross, mescaline

## Abstract

Current theories in the framework of hierarchical predictive coding propose that positive symptoms of schizophrenia, such as delusions and hallucinations, arise from an alteration in Bayesian inference, the term inference referring to a process by which learned predictions are used to infer probable causes of sensory data. However, for one particularly striking and frequent symptom of schizophrenia, thought insertion, no plausible account has been proposed in terms of the predictive-coding framework. Here we propose that thought insertion is due to an altered experience of thoughts as coming from “nowhere”, as is already indicated by the early 20th century phenomenological accounts by the early Heidelberg School of psychiatry. These accounts identified thought insertion as one of the self-disturbances (from German: “Ichstörungen”) of schizophrenia and used mescaline as a model-psychosis in healthy individuals to explore the possible mechanisms. The early Heidelberg School (Gruhle, Mayer-Gross, Beringer) first named and defined the self-disturbances, and proposed that thought insertion involves a disruption of the inner connectedness of thoughts and experiences, and a “becoming sensory” of those thoughts experienced as inserted. This account offers a novel way to integrate the phenomenology of thought insertion with the predictive coding framework. We argue that the altered experience of thoughts may be caused by a reduced precision of context-dependent predictions, relative to sensory precision. According to the principles of Bayesian inference, this reduced precision leads to increased prediction-error signals evoked by the neural activity that encodes thoughts. Thus, in analogy with the prediction-error related aberrant salience of external events that has been proposed previously, “internal” events such as thoughts (including volitions, emotions and memories) can also be associated with increased prediction-error signaling and are thus imbued with aberrant salience. We suggest that the individual’s attempt to explain the aberrant salience of thoughts results in their interpretation as being inserted by an alien agent, similarly to the emergence of delusions in response to the aberrant salience of sensory stimuli.

## Hierarchical Predictive Coding and the Positive Symptoms of Schizophrenia

Schizophrenia is typically characterized by a range of clinical features that comprise so-called *positive symptoms* such as delusions, hallucinations and self-disturbances, and *negative symptoms* such as reduced volition and affect expression. Current neurocognitive and computational theories propose that the positive symptoms of schizophrenia are due to an abnormality in the brain’s inference mechanisms (Corlett et al., 2009; Fletcher and Frith, 2009; Adams et al., 2013). Originating from Helmholtz’ idea of unconscious inference (Von Helmholtz, 1867), these theories conceptualize the brain as an inference machine that uses a predictive model to infer the (hidden) causes of the incoming sensory data (Dayan et al., 1995). This idea has been formalized in the framework of hierarchical predictive coding (Rao and Ballard, 1999; Lee and Mumford, 2003; Friston, 2005; Clark, 2013). The main idea is that a predictive model that represents knowledge and beliefs about the world serves to generate a stable and unitary perceptual experience from the inherently impoverished data registered by our senses. These predictions can be based in principle on any source of information other than the actual sensory stimulus that is subject to inference, that is, the stimulus whose cause is to be inferred. Predictions involve learned knowledge about the world as well as information from immediately preceding or concomitant events, i.e., contextual information (Clark, 2013; Phillips et al., 2015). Please note that we here use the term “context” in a broad sense as previously defined by Phillips et al. (2015), that is, referring to a wide range of phenomena that implicate predictive processing, such as contextual disambiguation, attentional processes, and learning. Predictive signals are thought to be fed back from higher to lower levels of cortical hierarchies. Whenever predictions are violated by sensory data, a prediction error signal is fed forward to the next higher hierarchical level to update the predictive model. Prediction errors thereby drive learning and enable a flexible adaptation to changes in the environment.

Computational approaches have conceptualized hierarchical predictive coding as Bayesian inference (Lee and Mumford, 2003; Friston, 2005). The underlying principle of Bayesian inference is that the probability of a prediction, which is commonly referred to as *prior belief*, is combined with the observed *sensory data* to compute a *posterior probability*. The difference between the means of the probability density functions of the prior belief and the sensory data corresponds to prediction error. Importantly, the size of the prediction error is weighted by the precisions of the prior belief and the sensory data: if the precision (which corresponds to the inverse of the width of the probability density function) of the sensory data is high relative to the precision of the prior belief, the precision-weighted prediction error will be greater and vice versa. It has been proposed that the psychosis is linked to decreased precision in the representation of prior beliefs relative to an increased precision in the encoding of the sensory data, which results in an abnormally strong weighting of precision error (Adams et al., 2013; Adams and Friston, in press); this can lead to the attribution of salience to otherwise irrelevant stimuli thus giving rise to delusional mood and the subsequent formation of delusions. This link between salience misattribution (or aberrant salience) and the formation of delusions was noted earlier by a number of authors, with or without direct reference to prediction error signaling as the underlying mechanism (Conrad, 1958; Heinz, 2002; Kapur, 2003; Corlett et al., 2009; Fletcher and Frith, 2009; Mishara and Corlett, 2009; Heinz and Schlagenhauf, 2010; Mishara, 2010b; Mishara and Fusar-Poli, 2013).

This predictive-coding account of altered inference has been related to the emergence of delusions of reference or persecution, and to a lesser extent delusions of control and hallucinations (Fletcher and Frith, 2009; Adams et al., 2013; Notredame et al., 2014), but not specifically to a group of positive symptoms that are commonly classified as *self-disturbances* (from the German “Ichstörungen”). In the present article, we propose an account within the predictive-coding framework of a particularly striking and frequently reported form of self-disturbance, the experience of one’s thoughts as being caused by somebody else, which is commonly referred to as *thought insertion*. In brief, we take our lead from the early Heidelberg School of phenomenological psychiatry and suggest that thought insertion is a form of aberrant or false inference, in analogy to the perceptual domain. Crucially, we will argue that the phenomenon of thought insertion entails a failure of hierarchical Bayesian inference to contextualize or predict the narrative interconnectedness of thoughts. In other words, there is an imprecise representation of context and a subsequent failure to provide top-down predictions of the neural representations of thoughts. These representations are therefore experienced as being caused by external forces, much in the same way that percepts are experienced as being caused by sensory input. It is this experience of thoughts as sensations that characterizes, as we indicate, thought insertion as a self-disturbance, in line with the phenomenological accounts of the early Heidelberg School. We will propose that the neurocomputational mechanism underlying this form of false inference is a reduction in the precision of prior beliefs relative to the precision in the encoding of thoughts, thus leading to increased prediction error and thus aberrant salience of thoughts. This account of thought insertion fits comfortably with explanations for delusions that are seen as a consequence of the aberrant salience of external stimuli, as outlined above.

## Thought Insertion and the Comparator Model: Can We Treat Thoughts like Actions?

The self-disturbances feature prominently among the first-rank symptoms proposed by Kurt Schneider. He described the self-disturbances as “certain disturbances in the experience of self that are highly specific for schizophrenia…[They] consist in the feeling that what one is and does is under the direct influence of others…[and] are found in the influencing of thought, feeling, impulse (drive) and will” (Schneider, 1946); modified English translation from Schneider (1959). Cutting (2015) observes that seven out of Schneider’s 11 first-rank symptoms, including thought insertion, are self-disturbances. Moreover, the remaining four are arguably related if we take the historical definition of the self-disturbances into account: “passivity—the nonparticipation in one’s own experience”, whereby one’s actions, cognitions, emotions, volitions, etc., are experienced as “occurring independently from self” (Schneider, 1959). As indicated in the subsequent section, the origin and history of the self-disturbances concept, however, beginning some 30 years prior to Kurt Schneider’s proposal of the first-rank symptoms, has remained nearly completely unexplored until only recently. As a result, commentators have almost universally propagated the assumptions that the self-disturbances are characterized by a “loss of a sense of mineness”, or “ipseity”, and that such self-disturbances are “core to schizophrenia” (Sass and Parnas, 2003; Zahavi, 2005; for reviews see Mishara, 2007, 2010a; Mishara et al., 2014). The actual originators and developers of the concept (namely, the earlier Heidelberg School as reviewed below) did not endorse either assumption. The lack of clarity concerning the history, concept and phenomenology of the self-disturbances has impeded the search for neurobiological mechanisms, which we attempt to correct in the current contribution. In this article, we focus on thought insertion, which refers to the experience of one’s own thoughts as caused by an alien agent.

The reasons for our interest in thought insertion are twofold. First, thought insertion is not only one of the most striking of the positive symptoms, but also a very frequent one occurring in approximately half of all patients diagnosed with schizophrenia (Sartorius et al., 1977) while absent in organic psychoses, e.g., those induced by tonic dopaminergic stimulation in Parkinson’s patients (Heinz et al., 1995). Second, there has been a long-standing debate regarding the relation of thought insertion to other positive symptoms with respect to current neurocognitive models and it has recently been questioned whether a Bayesian approach is able to provide a plausible account of thought insertion at all (Frith, 2012). Partially in response to the criticism (summarized below) that thoughts are not a kind of action, Frith abandons his earlier position that “the ‘made experiences’ including delusions of alien control and thought insertion, are associated with abnormalities in the mechanism that predicts the outcome of intended actions (the forward model)” (Frith, 2005). He (Frith, 2012) concedes: “a general, Bayesian approach does not provide a plausible account for the most striking of all first rank symptoms, *thought insertion*. This is ironic given that it was to explain this symptom that the comparator theory was first proposed”. Here we would like to argue that the previous problems in providing plausible accounts of thought insertion stem largely from the attempt to draw an analogy with delusions of control, in which not the thoughts but one’s own actions are experienced as being caused and controlled by an external agent. That is, we contend that it is possible to develop a Bayesian account of thought insertion without resorting to the analogy between thought and action. We do so precisely by relying on the earlier phenomenological work.

Delusions of control have long been proposed to result from a failure to predict one’s own actions due to impairment in generating “corollary discharge” signals (also called efference copy) that normally serve to attenuate the sensory consequences of self-initiated actions. The classic comparator model (Frith and Done, 1989) suggested that if the predicted and observed sensory consequences do not match, an action is experienced as generated by somebody else. This model can be translated into a Bayesian framework, in which corollary discharge would be equivalent to signaling Bayesian prior beliefs (i.e., a forward model of an action) and the sensory feedback signals correspond to the sensory data. Thus, similar to the idea that delusions of control result from a failure to attenuate the sensory consequences of one’s own actions (Frith and Done, 1989), the Bayesian account suggests a reduced precision in predicting these sensory consequences, resulting in increased precision-weighted prediction-error signals (Fletcher and Frith, 2009; Adams et al., 2013; Synofzik et al., 2013). It was recently noted, however, that even if the predicted and observed sensory consequences do not match, they will be attenuated during intended movements (Brown et al., 2013). Earlier accounts based upon the comparator and forward models failed to distinguish between the attenuation of precision during action and the suppression of prediction error *per se* by correct predictions or corollary discharge. This means that misattributions of agency and illusory phenomena characteristic of psychosis are better explained in terms of a failure to attenuate the precision of ascending sensory prediction errors. This failure of sensory attenuation can account for a resistance to illusions, such as the well-known force matching illusion (Brown et al., 2013).

In analogy with the comparator model, a mechanism on the basis of altered corollary discharge signaling was also proposed for thought insertion (Feinberg, 1978; Frith, 1992; Campbell, 1999), but this idea has been criticized for a number of reasons (Stephens and Graham, 2000; Gallagher, 2004; Vosgerau and Newen, 2007; Frith, 2012). Most critically, it has been questioned whether thoughts can be treated like actions. One key problem is that, in the case of motor acts, the comparator model implicates intentions for movement. The comparator functions to match the corollary discharge signal to the motor intentions, thus anticipating the movement and its sensory consequences. However, unlike actions, thoughts are not associated with intentions.^1^ In fact, the idea of having an intention to think leads to an infinite regress, whereby each thought would have to be preceded by an intention to think and this intention to think would also have to be preceded by an intention, and so on (Akins and Dennett, 1986; Stephens and Graham, 2000; Heinz, 2014). It has also been questioned whether a comparator mechanism is needed at all in the case of thinking (Gallagher, 2004). It is indeed implausible that the act of thinking should have sensory consequences that need to be “explained away” by a corollary discharge signal, as there is normally no need for distinguishing self-initiated thoughts from thoughts that are not self-initiated. Finally, the comparator model does not seem to sufficiently explain why inserted thoughts are experienced as being caused by another agent, whereas unbidden or intrusive thoughts that run through our heads unexpectedly or even against our will are not (Stephens and Graham, 2000; Gallagher, 2004; Vosgerau and Newen, 2007; Brewin et al., 2010; Heinz, 2014).

We concur with the previous critiques in that we do not find the analogy between thoughts and actions particularly helpful. Instead, we suggest that the flow, or continuity of thinking relies much more on the automatic processing of context—including previous thoughts—than we are usually prone to admit or observe.^2^ In the next section we provide a historical introduction to the concept of self-disturbances, and in particular thought insertion, with an emphasis on the early 20th century phenomenological accounts of these symptoms. Interestingly, these phenomenological descriptions highlighted thought insertion as a “perceptual experience”. On the basis of these observations, we then go on to develop an account of thought insertion within the framework of hierarchical predictive coding and Bayesian inference, which suggests that the prediction errors may be more “low-level” than current theories of thought insertion would suggest.

## The Phenomenology of Thought Insertion

In the literature, the origin of the term self-disturbances is frequently attributed to Jaspers (for review see Mishara et al., 2014). Although Jaspers (1913) described phenomena related to self-disturbances, such as passivity experiences, being influenced, inserted or withdrawn thoughts, he did not systemize them under the concept of self-disturbances. The historical reviews then jump to Schneider’s systemization of the self-disturbances in the 1940s, which are then included among the first rank symptoms, thus omitting not only who coined the term but nearly 30 years of its development. Only recently, Mishara et al. (2015) documented for the first time (as far as we know) the role of Jaspers’ Heidelberg colleagues (the early Heidelberg School, i.e., Gruhle, Mayer-Gross, and Beringer) in the origin and development of the self-disturbance concept (see Hermle et al., 1988 for exception in their acknowledgment of Beringer’s contribution). In fact, it was Gruhle (1915) who coined the term self-disturbance: “Tentatively, I call this passivity—the nonparticipation in one’s own experience—a self-disturbance” (our translation). In coining the term, Gruhle (1915, 1922, 1929) identified two components of self-disturbances: a perceptual-component, whereby the self looks passively on its own perceptual experiences as a non-participatory bystander, in a *doubling/splitting* of the I (Doppel-Ich), as if everything plays before the subject on a theater-stage; and a higher-cognitive component, implicating that thoughts, feelings, actions and volition are experienced as coming under foreign power(s). Not recognized as double(s), the foreign agents have power over the self.

The further development of the concept of self-disturbances was closely linked to a study by Heidelberg psychiatrists Beringer and Mayer-Gross in the 1920’s, which used mescaline to examine the phenomenology of psychotic experiences, especially the self-disturbances, in healthy participants. The participants included psychiatrists and medical students with the goal of becoming more able to empathize with or understand those experiences of psychotic patients that Jaspers (1913) had labeled as non-understandable due to an underlying as yet unknown neurobiological disease process, which interrupts the development of the person. On the basis of his experiences as a participant in the mescaline studies, Mayer-Gross noted that thoughts are not *ascribed* to alien agency, but that they are rather *perceived* as alien. He described a “becoming sensory” (Versinnlichung) in the “sensory representation of thoughts…Without this change, the manifestations remain inexplicable” (Mayer-Gross and Stein, 1928; Mayer-Gross, 1932). He further notes that we obtain “insight into the progressive invasiveness of the thought-disturbances from the earliest sign of difficulties in concentrating […]. These prodromal manifestations lead to a scattered emptiness of thinking, quite similar to thought withdrawal” (our translation). In his careful phenomenological analysis of subtle self-perceived cognitive and other disturbances in prodromal schizophrenia and mescaline intoxication, Mayer-Gross anticipated Huber’s basic-symptom concept (Huber, 1995; Mishara et al., 2015).

As far as we know, Mayer-Gross’ novel hypothesis that thought insertion involves a “becoming sensory” of individual, intermittent thoughts has not been previously discussed in the secondary literature on thought insertion or other self-disturbances. It also seems to have escaped the attention of commentators that Mayer-Gross is the herald of what later came to be known as the “perceptual anomalies” approach to the positive symptoms of schizophrenia, the view that low-level perceptual anomalies play a critical role in the positive symptoms of schizophrenia, including thought insertion and other self-disturbances. The phenomenological psychiatrists Matussek, Conrad and Binswanger later developed this view (for reviews see Uhlhaas and Mishara, 2007; Mishara, 2010b; Mishara and Fusar-Poli, 2013). Later, others came to similar conclusions (Maher, 1974; Frith, 1979; Gray et al., 1991).

The observations from Beringer’s and Mayer-Gross’ mescaline experiments supported Gruhle’s earlier observations concerning the self-disturbances on several points. First, they supported Gruhle’s (1915, 1922, 1929, 1932) proposal that perceptions, movements, hallucinatory experiences, feelings, volitions, remembering, speaking and thinking are experienced in the self-disturbances as having “independence from self” (Beringer, 1927). Generally, we experience controlled and automatic processing working together seamlessly in everyday cognition, without giving much thought to how this takes place. In self-disturbances, however, the patient experiences these underlying automatic processes as independent “automatisms” having foreign origin.

Second, Beringer and Mayer-Gross concur with Gruhle’s argument that thought insertion and other self-disturbances are non-understandable in Jaspers (1913) sense, as they cannot be derived from previous content or understandable motivation in the patient’s personality. Gruhle observed that inserted thoughts often concern harmless, mundane circumstances. Despite an absence of obvious difference in *content* from other thoughts, the patient knows precisely which thoughts are inserted. Recently, we (PS, ALM) interviewed a schizophrenia patient who describes inserted thoughts, in line with Gruhle’s observation, as not particularly unusual in content. For example, the patient ascribes his (perfectly mundane) thought to go to the supermarket and buy bananas to someone else thinking the thought, because he cannot derive it from the current context of his thinking: “It seems that someone else is in the supermarket and thinks, ‘I will now buy bananas’. Then I have this thought in my head, although I was not thinking about the supermarket. […] What is disturbing is not the content, but the constant interruption of my thinking.”

Third, Gruhle, Beringer and Mayer-Gross agree that self-disturbances (Ichstörungen) generally involve the interruption of understandable context which requires an inner connectedness of experience. This includes how thinking is anticipated moment-to-moment as continuous. Beringer writes, “In healthy individuals there is the always unconscious heading towards a totality, an intuitive knowing where the thought is going, which is missing in thought-disturbances” (Beringer, 1924). Seeming to come from “nowhere”, [Gruhle’s phrasing] inserted thoughts disrupt the on-line contribution from just past thoughts in shaping the inner continuity and goal-directedness of thinking (Gruhle, 1922, 1929). Due to the ongoing interruption by inserted thoughts and “made” perceptions or movements, there is a reduction of what the patient anticipates (“das Vorschauende”); there is only the compelling sensory-evidence of now: “No temporal order prevails, each sensory impression is equally valued, replacing its predecessor” (our translation; Mayer-Gross and Stein, 1928).

Finally, Gruhle (1922, 1929, 1932) and Mayer-Gross (1932) agree that the self-disturbances should be counted among the “primary symptoms” of schizophrenia (in Jaspers, 1913 sense). That is, *the primary symptoms may co-occur but are independent (orthogonal) and are not specific, nor core to schizophrenia*.

Despite points of agreement, Mayer-Gross and Gruhle disagree on the role of low-level sensory processing in generating thought insertion and other self-disturbances. Gruhle concludes that the underlying perception remains intact. He reasons that if one listens to patients who state that something is conspicuous or salient in their perceptual environment, even the most articulate patients are unable to say precisely what they actually find striking. Mayer-Gross counters that such arguments are not based on phenomenological evidence but rather on an absence of evidence, from which one cannot draw conclusions. For Mayer-Gross (Mayer-Gross and Stein, 1928; Mayer-Gross, 1932), the clinical interview is key in ascertaining the phenomenological data by freeing ourselves “from theoretical prejudices and the need to derive everything from the content of these experiences” (our translation), i.e., the “what” as opposed to the “how” of these experiences (see immediately below). The early Heidelberg School’s views on thought insertion were developed on the basis of Jaspers (1913) method of attempting—during clinical interview—to understand or empathize with the patient by reconstructing from moment to moment the inner, subjective connectedness of patients’ experience and thoughts. We note that the phenomenological definition of thought insertion and the self-disturbances as loss of context (here the interconnectedness of thoughts in subjective time) dovetails nicely with the predictive coding approach (see Giersch and Mishara, in press). Thought insertion involves a disruption in the patient’s on-line continuity of thinking that impedes the interviewing clinician’s *empathic understanding* or *reconstruction of context* in Jaspers’ sense (for Jaspers’ method, see Mishara and Fusar-Poli, 2013). For Mayer-Gross, the mescaline experiments were critical in that they “opened the way to explaining these non-empathizable forms of subjective experience, which, up till now, have occasioned very unsatisfactory efforts to interpret or *theoretically* understand these experiences” (our translation; Mayer-Gross and Stein, 1926).

In his assertion that thought insertion is thinking “becoming sensory”, Mayer-Gross not only contends with Heidelberg colleague, Gruhle, but also encounters opposing views in his contemporary, Leipzig-based psychiatrist, Paul Schröder. For his theory of thought insertion, Schröder leans heavily on the celebrated neuropsychiatrist Wernicke. Both Wernicke (1906) and Schröder (1915, 1921a,b, 1926, 1928) argue that thoughts becoming loud (Gedankenlautwerden), auditory verbal hallucinations, thought insertion and “the made thoughts and experiences” (Wernicke’s autochthonous ideas) are not based on a perceptual disturbance but strictly on a verbal-linguistic one (i.e., “phonemes”). For this reason, Schröder proposes that all these symptoms are related in content and may transition from one to the other during the course of the illness in schizophrenia. He finds thought insertion is often the precursor in the course of illness to thoughts becoming loud and auditory verbal hallucinations. Schröder concludes that thought insertion, thoughts becoming loud and auditory verbal hallucinations are related symptoms and groups them under the unitary concept “verbal hallucinosis”.

Despite the emphasis on phonemes, he proposes that the critical element in this symptom-complex is a “feeling of foreignness” (Fremdheitsgefühl). He regards the “feeling of foreignness” as primary, a not further analyzable or explainable original phenomenon. Its imposition on the phonemes gives them their foreign character, an “externalizing” of thoughts. The patient often thinks that the made or influenced thoughts are caused by hypnosis, suggestion, etc., i.e., delusional explanations (Erklärungswahn). However, each of these symptoms result from “transitions” and variations in the fundamental “feeling of foreignness”. Progressing from thought insertion to thoughts becoming loud and auditory verbal hallucinations, they involve “a co-speaking, a speaking before, a speaking after, a repeating, or an answering back” (Schröder, 1928).

Mayer-Gross (Mayer-Gross and Stein, 1928; Mayer-Gross, 1932) counters that Schröder’s “feeling of foreignness” (Fremdheitsgefühl) as the not further analyzable original phenomenon of verbal hallucinosis is theoretical and thus, arbitrary, i.e., not based on phenomenological method or data. He comments: “To this day we know nothing of the inner interconnectedness of these symptoms and claims *about their essence* are presumptuous. Rather, by sticking faithfully to patients’ reports of subjective experience, we consistently find descriptions which clearly go back to the sensory sources…the perceptual anomalies in beginning schizophrenia” (Mayer-Gross, 1932). According to Mayer-Gross, what makes the inserted thought stand out from other thoughts is not a “feeling of foreignness,” but rather *the experience of individual thoughts becoming sensory*. This involves a transformed sense of what is experienced as perceptual according to a “functional transformation” (Mayer-Gross and Stein, 1928; Beringer and Ruffin, 1932; Weizsäcker, 1950). That is, the way that the perception is given is radically altered, and is “incomparable” to previous perceptual experiences (Mayer-Gross and Stein, 1928; Mayer-Gross, 1932). *Importantly, what makes thought insertion and other self-disturbances aberrantly salient is not the content of the thoughts, volitions, etc., but the manner of their givenness, not the “what” but the “how” of the experience*. Thus patients do not infer that their thoughts, hallucinations, etc., are caused by foreign agency from the content but from *how* they are given, as radically altered, “incomparable” to previous cognitive and perceptual experiences. It is likely that Mayer-Gross developed this approach not only from the acuity of his clinical observations, but from his own insights directly resulting from his mescaline experience.

Counter to Schröder’s thesis of a successive progression of related symptoms from thought insertion to thoughts becoming loud and then, auditory verbal hallucinations, Mayer-Gross observes in some patients the nearly simultaneous experience of akoasm (nonverbal auditory hallucinations, e.g., buzzing, whistling, roaring), auditory verbal hallucinations, and thought insertion. Importantly, they are not interpreted as belonging to one symptom complex (as Schröder’s “verbal hallucinosis”), but orthogonal occurrences which nevertheless may involve similar neural mechanisms. The voices and akoasms “surprise” and thought insertion “interrupts”, but they all involve the disruption of context according to a “functional change” of the lower-level sensory basis of these disturbances, which cannot be surmised by a generalized “feeling of foreignness” (Mayer-Gross and Stein, 1928; Mayer-Gross, 1932). The latter is merely a change in content (“what”) but not “how”, the modality in which the experience is given.^3^ The parallel occurrences of these symptoms indicate different levels of disruption of the Gestalt organization of the experience, a point later developed by Matussek (1952, 1953), Conrad (1958) and Binswanger (1965) (for reviews see Uhlhaas and Mishara, 2007; Mishara, 2010b, 2011).

## Thought Insertion as a Consequence of Reduced Precision of Prior Beliefs

A central aspect of the phenomenological accounts of self-disturbances is that an altered experience of thoughts is at the core of thought insertion as a self-disturbance. Because inserted thoughts are perceived as especially surprising and hence not in continuity with previous thoughts, they are experienced as coming from “nowhere” and interpreted as being inserted by somebody else. Here we propose that, in analogy with the aberrant salience of external events that leads to the emergence of delusional beliefs (Heinz, 2002; Kapur, 2003; Corlett et al., 2009; Fletcher and Frith, 2009; Heinz and Schlagenhauf, 2010; Mishara and Fusar-Poli, 2013), internal events such as thoughts may also be experienced as overly salient and therefore unusual and surprising. The individual’s attempt to explain the aberrant salience and unusual character of thoughts (what Mayer-Gross identifies as “how” they are given) results in their interpretation as being caused by an alien agent. To provide a plausible account of thought insertion within the framework of hierarchical predictive coding, two key questions need to be addressed. In the first place, how can the aberrant salience of thoughts be accounted for by altered Bayesian inference? And in the second place, if aberrant salience is at the core of thought insertion, why should it result in the experience of thoughts as being caused by an external agent? We address these two questions in turn.

### Reduced Precision of Prior Beliefs and Decontextualization

With regard to the first question, we propose that the mechanism underlying aberrant salience of thoughts is reduced precision of prior beliefs about these very thoughts. As discussed previously by Martin and Pacherie (2013), thoughts are normally experienced as our own thoughts because they are embedded in a context. As they put it, we experience our thoughts as our own because of “online dynamical processes of causal–contextual information integration”. This contextual information comprises external stimuli (“I’m thinking about Ada because I just saw her photo”) or preceding thoughts (“I’m thinking of Ada because I just thought of her sister Cosima”), but also explicit or implicit beliefs, emotions, desires and interests (Campbell, 1999; Stephens and Graham, 2000; Martin and Pacherie, 2013). In terms of predictive coding, such contextual information is the source of prior beliefs (or predictions) about upcoming thoughts. Importantly, we propose here that this predictive context need not be conscious: I may think about Ada without knowing why, but it does not bother me because the context is still present unconsciously. This is consistent with phenomenological accounts of unconscious automatic processing providing context to expect what comes next (Gruhle, 1922, 1929). The failure to integrate contextual information results in a “decontextualization”, (what the phenomenological psychiatrist Blankenburg called a “context blindness” or a loss of common sense; Blankenburg, 1984; Blankenburg and Mishara, 2001) which in turn may lead to an altered experience of thoughts as surprising and as a consequence, their attribution to an alien agent.

The failure to integrate contextual information such as reference to a preceding thought could also be linked to symptoms such as loose word associations, incoherent speech and deficits in abstract thinking that characterize formal thought disorder in schizophrenia. As a result of loosened associations, several studies show alterations in tests of word association (for a review see Spitzer, 1992) or semantic priming (e.g., Moritz et al., 2003). In such tasks, unconscious automatic spread of activation within semantic memory in schizophrenia patients is increased and farther-reaching than in healthy individuals, resulting in co-activations of semantically related concepts, which in turn affects goal-directed thinking. One could speculate that such abnormal associative processes are involved in the occurrence of inserted thoughts in schizophrenia: arbitrary stimuli might trigger an association chain that goes far beyond the normal scope. The resulting thoughts may be experienced as coming from nowhere, since the trigger cannot be traced back anymore, even implicitly, as non-conscious context. In order to explain the unusual experience, the individual may form a delusional explanation for the occurrence of the untraceable thought.

The notion of disrupted context integration is supported by a large body of experimental evidence for altered context processing in patients with schizophrenia. For example, psychophysical studies investigating visual illusions showed that the influence of spatial context in the perception of visual stimuli is reduced in patients with schizophrenia. The perceived contrast of a central stimulus is normally reduced when embedded in a high contrast surround, a phenomenon called surround suppression (Chubb et al., 1989). Patients with schizophrenia are less susceptible to this effect: under conditions that induce such surround suppression in healthy individuals, patients with schizophrenia have a more veridical perception of the center contrast than controls (Dakin et al., 2005; Yoon et al., 2009, 2010; Barch et al., 2012; Gold et al., 2012; Tibber et al., 2013; Yang et al., 2013) and show a reduced modulation of neural responses already at the lowest stages of cortical visual processing (Seymour et al., 2013). Reduced context effects were also reported for motion and size perception (Uhlhaas et al., 2004; Tadin et al., 2006; Uhlhaas et al., 2006). It should be noted, however, that release from contextual suppression in schizophrenia patients is not uniformly found for all visual context effects (Tibber et al., 2013; Yang et al., 2013) and that there is only limited evidence for correlation with symptom severity. The latter point may be due to relatively small sample sizes in some of these studies (Tibber et al., 2013; Yang et al., 2013) or to samples of relatively stable patients with a limited range of psychotic symptoms (Gold et al., 2012). Alternatively, the lack of correlation may point to reduced context effects as a trait rather than a state marker of psychosis, as discussed earlier (Seymour et al., 2013). To our knowledge, none of these studies specifically investigated the relationship between altered visual context processing and thought insertion. Similar to spatial context, effects of temporal context on visual perception are also reduced in patients with schizophrenia, as indicated by a reduced influence of preceding perceptual states on the current perception of ambiguous visual stimuli (Schmack et al., 2015). Interestingly, this latter finding was found to correlate with the severity of delusions (Schmack et al., 2015) and with delusion-proneness in healthy individuals (Schmack et al., 2013). Again, whether such temporal context effects are also related to thought insertion remains to be investigated. These perceptual phenomena are characterized by a high degree of automaticity and resistance to cognitive influence, which is why they are thought to be implemented at low levels of the visual processing hierarchy, i.e., within sensory cortices. The altered perception of visual illusions and ambiguous stimuli in schizophrenia thus suggest a functional abnormality at low hierarchical levels, thus pointing to a pervasive deficit that involves predictive processing throughout all levels of the predictive coding hierarchy. This idea is in line with observations by various phenomenologists (including Mayer-Gross, Binswanger and Conrad), who concluded that the anomalies enter at a very early stage of sensory processing. In addition to these findings in the domain of perception, there are also examples of altered learning in patients with schizophrenia. One such example is latent inhibition, a phenomenon whereby a stimulus that has been presented repeatedly, but has not had any predictive value is difficult to associate with an outcome at a later stage. In other words, the context of previous stimulus exposures (in which no predictive value was associated) influences inference with regard to this stimulus later in time (Fletcher and Frith, 2009). In individuals with schizophrenia, latent inhibition is reduced, that is, they learn to associate stimuli to which they have been previously exposed with a predictive value more easily than healthy controls (Vaitl et al., 2002). Another example of reduced context effects in learning is the observation that in people with schizophrenia the recall of extinction learning following fear conditioning is impaired, with a reduced effect of the context that was learned during extinction on conditioned responses (Holt et al., 2009).

In contrast to the special case of visual illusions, a deficit in context integration will not alter the actual content of conscious perception in everyday vision, but may nevertheless result in the aberrant salience of sensory stimuli. Similarly to the processing of external sensory events, a context integration deficit may also play a role in the process of thinking (Martin and Pacherie, 2013) and imbue thought with aberrant salience that disrupts the inner continuity of experience as described by the earlier phenomenologists (Gruhle, 1922, 1929, 1932). Additionally, “concrete” misinterpretations of proverbs by patients with schizophrenia may be explained as a failure to integrate context information that usually guides their automatic interpretation. Proverbs are instead interpreted “word-by-word”, that is, according to the literal meaning of the words rather than in an abstract sense (Heinz, 2014). As indicated above, the phenomenological psychiatrists Mayer-Gross and later Binswanger, Blankenburg, Conrad and Matussek pointed to a loss of context as fundamental to delusional-formation and the self-disturbances on different levels of perceptual processing (Mishara, 2011; Mishara and Fusar-Poli, 2013).

In terms of Bayesian predictive-coding, contextual information for thoughts can be seen as constituting prior beliefs as to which thoughts are likely to occur next. Moreover, we expect thoughts to behave like thoughts and not like “perceptual objects,” which seem to appear from nowhere. If the precision of these prior beliefs is reduced and/or sensory precision is increased, this will lead to an increase in the precision-weighted prediction error evoked by the neural activity that encodes these thoughts. Such poorly predicted thoughts will be experienced as aberrantly salient, just as poorly predicted perceptions are, and will evoke an experience of surprise. We propose that it is the aberrant salience caused by increased precision-weighted prediction error that is at the core of the “strange feeling” that patients report to be associated with inserted thoughts (Vosgerau and Newen, 2007). Unlike Schröder’s “feeling of foreignness,” which is stated as something ultimate, as not further analyzable, or explainable, the “strange feeling” here is neither ultimate nor final. The “feeling of foreignness” is, in Mayer-Gross’ terms, a content rather than a mode of givenness (Mayer-Gross and Stein, 1928; Mayer-Gross, 1932) and thus in itself not contributing to its experience as sensory, which, in our view, rests on mechanisms modeled by predictive coding.

Importantly, when conceiving of thoughts as being predicted by prior beliefs that are determined by context information, we make no principled assumption as to whether this context information is consciously accessible. In Bayesian inference, a prior belief is considered merely a probability distribution over some unknown state and may or may not be consciously accessible (Adams et al., 2013; Mishara and Sterzer, 2015). Importantly, current models of hierarchical predictive coding assume that prior beliefs are fundamentally embodied even at the lowest levels of sensory processing (Friston, 2005; Adams et al., 2013). For instance, most of the above-mentioned influences of context (both spatial and temporal) on visual perception, which are prime examples of efficient predictive coding (Clark, 2013), take effect automatically and are often not accessible to conscious introspection. Similarly, context-based prior beliefs about thoughts may be implemented automatically without the individual’s awareness, similar to the above-mentioned example of proverb interpretation. This view is also supported by neuroimaging work suggesting neural representations of upcoming decisions about mental operations that can be decoded several seconds before an individual becomes aware of the decision (Soon et al., 2008). It is thus conceivable that prior beliefs about upcoming thoughts do have a neural representation that is not necessarily accessible to introspection. Yet, a reduced precision of such a representation (also unconscious) may result in an increased precision-weighted prediction error associated with neural activity underlying a subsequent thought, thus imbuing the thought with the qualities normally associated with sensations, and thus aberrant salience. A first step in putting this idea to the test empirically would be to assess the relationship between thought insertion and altered sensory context processing. We would expect that reduced precision in the signaling of predictive context would be associated with an increased occurrence of thought insertion. The direct empirical investigation of our hypothesis that thought insertion is due to increased prediction-error signaling associated with the neural events underlying thoughts is an intriguing challenge for future research and will require the development of new experimental paradigms. For example, one could investigate the effects of conscious and unconscious contextual information on the experience of one’s own thoughts using the modulation of the predictive context by (supra- and subliminal) priming. Furthermore, computational modeling could be used to estimate the parameters for the precision of predictions and for precision-weighted prediction errors, thus linking thought insertion to the neural computations underlying predictive coding (see Iglesias et al., 2013; Schmack et al., 2016).

The view outlined here has important implications for the question whether intentions to think play a role in thought insertion (Akins and Dennett, 1986; Stephens and Graham, 2000; Heinz, 2014). The hierarchical predictive-coding account of thought insertion has the potential to solve this problem as it does not invoke intentions to think but relies on prior beliefs about thoughts and how thoughts are supposed to behave, which may or may not be consciously accessible. In a similar vein, the view proposed here also has implications for the distinction between inserted and unbidden thoughts (Stephens and Graham, 2000; Gallagher, 2004; Vosgerau and Newen, 2007; Heinz, 2014; Vosgerau and Voss, 2014). Both may be experienced as coming from nowhere, meaning that in both cases the occurrence of a particular thought at a particular time may be surprising to the individual who in both cases may be unable to introspectively explain the occurrence of the thought. Vosgerau and Voss (2014) therefore distinguish *control* over thoughts (which is disturbed in both, inserted and intrusive/unbidden thoughts, respectively) from *authorship* of thoughts (which is disturbed in the case of inserted thoughts only). The fact that only inserted thoughts are attributed to an alien agent may be accounted for by the aberrant salience of such thoughts. Again, the cause of aberrant salience, low precision of prior beliefs and/or increased sensory precision, may not be accessible to introspection, leaving the strange feeling associated with inserted thoughts unintelligible to the individual. While beliefs and desires can have a role in the prior beliefs regarding our own thoughts (Stephens and Graham, 2000), what makes thoughts feel alien is not the result of a higher-level introspection but rather the prediction-error based aberrant salience caused by imprecise prior beliefs. It is therefore not the failure of introspection in a comparator process (Campbell, 1999) but rather the failure (or imprecision) of prior beliefs in a Bayesian inference process that is at the core of thought insertion.

### Aberrant Salience of Thoughts and Their Attribution to an Alien Agent

We have now outlined how a thought that is poorly predicted by prior beliefs can evoke aberrant salience in analogy with sensory events that are associated with excessive precision-weighted prediction error. The question remains whether this aberrant salience is sufficient to explain why patients experience such thoughts as inserted by an alien agent. Here we argue that aberrant salience provides a sufficient explanation for the experience of thought insertion. To do so, we first turn to the question what it is that normally makes us experience our thoughts as being generated by ourselves and belonging to ourselves, which is often referred to as sense of authorship or sense of ownership, respectively. It has been proposed that thought insertion reflects a disrupted sense of ownership, and as a consequence also a disturbed sense of authorship (for discussion see Martin and Pacherie, 2013; Vosgerau and Voss, 2014). One non-trivial problem with this idea is the assumption that we have a pre-reflective self-awareness which imbues each experience with a sense of “mineness”. This basic sense of mineness (also called “ipseity”) supposedly accompanies every experience and becomes the condition of having any experience at all (e.g., Henry, 1973; Zahavi, 2005); for reviews see (Mishara, 2007, 2010a; Mishara et al., 2014). However finding reflective (phenomenological) access to the putative pre-reflective mineness poses considerable methodological challenges. Moreover, these challenges of accessing pre-reflective consciousness as a pure (irrelational) “passive self-affection” (Henry, 1973, 1989, 2003; Zahavi, 2005) should give us pause from simply identifying this evasive, but putatively all-pervasive quality with either consciousness or interoception. The latter involves a perception action cycle, i.e., an experienced content. Notably, ispeity cannot be the content of a perception, including interoception, because this would objectify it. Rather, ipseity is seen as the condition for, and therefore, must “precede” any perception action cycle for the latter to take place at all. This position, however, neglects how rapidly and automatically such perception action cycles may occur and any “access” to ipseity would be comparatively “slower” because it would require a retrospective reflection or verbal report (and thus unable to rule out so-called observer or verbal overshadowing effects) (see Mishara, 2010a; Mishara et al., 2014)^4^. Moreover, as Vosgerau and Voss (2014) point out, the content of an experienced thought does not necessarily contain self-reference. It thus remains moot whether the idea of pre-reflective self-awareness as a pervasive feeling of mineness (so-called ipseity or pure “passive self-affection”) is what is disrupted in thought insertion. Instead, based on the phenomenological accounts by the early Heidelberg school (see above), we propose that it is not that something is merely missing but rather something added or different about the experience of thinking. The description of thoughts as “becoming sensory” (Mayer-Gross) in the interruption of context and inner connectedness of experience (Gruhle, Beringer) does not indicate the absence of a feeling of mineness, but rather an altered modality of thoughts, now given as sensory objects, and thus, a disruption of context.

Here, we therefore suggest that sense of authorship and sense of ownership are not generated by some specialized neural circuitry that produces an experience of “mineness” or a “feeling of authorship”. Rather, they are a result of thoughts and actions being predictable on the basis of the current contextual information, as described above. That is, if a thought is predicted by (conscious or unconscious) contextual information and thus matches prior beliefs, we experience this thought as generated by ourselves and “belonging” to us. This is also compatible with the phenomenology of automatic processing of contextual meaning (Gruhle, 1922, 1929; Mishara, 2007, 2011). This is normally the case, that is, the “default” manner of thinking is that thoughts are predictable. That is, there is an on-line predicting of the inner continuity of one’s thoughts and experiences on different time scales—some conscious, some not conscious—which serves as the context for this default view concerning our thoughts. If the precision of prior beliefs is abnormally low, and the posterior belief is biased towards the precision of sensory evidence (as the mode of givenness of the thoughts), however, the thoughts may be associated with aberrant salience. As it does not occur normally, such salience is an unusual and surprising event, which requires some explanation. According to Vosgerau and Newen (2007), this misattribution of thoughts to an alien agent is actually “a healthy and normal rationalization”. They suggest that if there is a conflict between beliefs and perceived states, this conflict is removed by some form of rationalization; and that a quite normal way of rationalizing is to misattribute the cause of the perceived state. In the case of thought insertion, it is “some kind of strange feeling about one’s own thoughts” that creates the dissonance to be rationalized (Vosgerau and Newen, 2007). In a way, this may be alike to the situation in which one’s own thoughts are interrupted by the utterance of another person—it is unexpected and hence to some degree surprising, and thus a totally unexpected thought may appear to come from someone else as the most likely explanation of its surprising character.

Here, Martin and Pacherie (2013) proposed that the attribution of thoughts to an alien agent is the result of an attempt to make sense of this strange experience of having a thought that appears out of context. Thus, they suggest that the interpretation of one’s own thoughts as being inserted by someone else is an attempt to “recontextualize” thoughts that seem to come out of nowhere. The recontextualization or rationalization of thoughts as being inserted by an alien agent of course appears bizarre, but as Martin and Pacherie (2013) put it, “extraordinary events call for extraordinary explanations”. In other words, the strange experience that we suggest reflects prediction-error-based aberrant salience of thoughts is an unusual event that does not normally occur (as the default experience is that thoughts are predictable and not given as interrupting sensory experiences). Even if the interpretation that such thoughts are inserted by an alien agent appears bizarre, it may in fact be the most plausible explanation for thoughts that are associated with aberrant salience. In addition, there may be a readiness to attribute the cause of such thoughts to another agent on the basis of delusional mood in prodromal period (prior to fully formed self-disturbances) or, following conversion to full-blown psychosis, and already established delusional beliefs (see Conrad, 1958; Mishara, 2010b). If a propensity towards bizarre explanations for one’s surprising experiences has already developed, then the interpretation of surprising thoughts as caused by another agent (which one would normally reject as bizarre) may be more readily accepted. Alternatively, the alien agency may be, as Mayer-Gross suggests, the thoughts are not *ascribed* to alien agency, but are rather directly *perceived* as alien (Mayer-Gross and Stein, 1928; Mayer-Gross, 1932). Nevertheless, hierarchical predictive-coding is consistent with both alternatives and provides a way to integrate them into one account of thought insertion.

## Author Contributions

PS and ALM: conceived the contents and structure of the article. PS, ALM, MV and AH: co-wrote the article.

## Funding

This project was funded by The Chicago School of Professional Psychology.

## Conflict of Interest Statement

The authors declare that the research was conducted in the absence of any commercial or financial relationships that could be construed as a potential conflict of interest.

## Footnotes

^1^Conversely, beginning meditators who practice mindfulness anecdotally report that the “intention” not to think brings little relief to the deluge of thoughts that follow. This suggests that thinking is not easily guided by “intention” in either producing thoughts or resisting them. Note that “thinking” here is not confined to the deliberate, effortful search which, for example, accompanies such phenomena as the “tip of the tongue.”

^2^By emphasizing agency and intention, the role of automaticity in thinking has often been neglected. To take one example, which at least indirectly suggests an automatic component, the philosopher Heidegger (1969, 1977) claims that “thinking” is neither the product of the agency of an “I,” nor the relationship of ownership to what is thought. Rather, thinking is a “letting be” of the matter thought about, which, in turn, discloses itself, opens itself to thought, as if thinking itself were a conversation, a dialog of question and answer which is future oriented. The “opening sets us the task of learning from it, while questioning it, that is, of letting it say something to us” (Heidegger, 1977). We do not enter here into Heidegger’s ontological and philosophical goals, which are not critical to our argument. As the Heidelberg psychiatrist, Gruhle (1922, 1929, 1932) notes, the “medium,” who, in a state of “possession” imparts the thoughts of the spirit possessing her does not experience thought insertion. The medium is conscious of her role as medium, and even if the possession is against her will, it does not lead to thought insertion. One may be “surprised” by one’s own thoughts and even learn from them, but does not experience these thoughts as inserted because they occur within a more or less expected context.

^3^It is important to note that in his application of phenomenological method, Mayer-Gross not only countered Schröder’s “feeling of foreignness”, but also psychoanalytic approaches at the time which based their theoretical explanations on content. On first glance, it would seem that Mayer-Gross’ novel hypothesis of thoughts becoming sensory in thought insertion resembles Freud’s own account of words (thoughts) becoming replaced by symbolic images in dreams, hypnagogic images or psychosis as a kind of rebus (or puzzle), i.e., what Freud calls a “topographical regression” from word-presentations (Wortvorstellungen) to thing-presentations (Dingvorstellungen). In both the phenomenological and psychoanalytic accounts, thoughts start to behave like perceptual objects. However, the phenomenology of thought insertion (and its implicated mechanism) is not based on content (which can be *understandably* derived from the patient’s past personality, motivations, unconscious wishes, Weltanschauung, etc., as in psychoanalysis), but rather on its “mode of givenness”, not its “what”, but its “how”. That is, the patient is able to identify which thoughts are inserted not in terms of their content, which may not be unusual, but rather how they are given (i.e., experienced as sensory objects).

^4^Nevertheless, the “ipseity” concept is a moving target because it has not been fixed by one definition, nor completely operationalized - and perhaps, in principle, cannot be operationalized—leading to adjustments of the concept over time to fit in with other theories, or objections.
